# Multi-strata “camera columns”: an effective approach to characterize non-volant mammal communities in tropical forests

**DOI:** 10.1093/jmammal/gyag047

**Published:** 2026-05-29

**Authors:** Daniel Alempijevic, David Essian, Charlene Fournier, Martin Balimu, Junior Amboko, Kate M Detwiler

**Affiliations:** Department of Biological Sciences, Florida Atlantic University, 777 Glades Rd, Boca Raton, FL 33431, United States; Harte Research Institute, Texas A&M University, 6300 Ocean Drive, Corpus Christi, TX 78412, United States; Department of Biological Sciences, Florida Atlantic University, 777 Glades Rd, Boca Raton, FL 33431, United States; Agronomic Sciences: Fauna and Flora, Kindu University, Site de Lwama, Kindu, Maniema, Democratic Republic of the Congo; Department of Biological Sciences, Florida Atlantic University, 777 Glades Rd, Boca Raton, FL 33431, United States; Department of Biological Sciences, Florida Atlantic University, 777 Glades Rd, Boca Raton, FL 33431, United States

**Keywords:** arboreal mammals, camera trapping, community ecology, Congo Basin, Lomami National Park, multi-strata sampling, species richness, tropical forests, vertical stratification, Bassin du Congo, échantillonnage multi-strates, écologie des communautés, forêts tropicales, mammifères arboricoles, Parc national de la Lomami, piége photographique, richesse spécifique, stratification verticale

## Abstract

Understanding how arboreal mammals partition vertical space is essential to accurately characterize tropical forest communities, yet most camera trap surveys focus on the forest floor, or pair canopy and ground surveys. While canopy camera trapping is revealing new insights about arboreal mammals, sampling at a single height at each sampling point may obscure patterns in community structure. To address this gap, we developed a “camera column” technique, deploying 3 vertically aligned cameras at ground, understory-midstory, and canopy levels across 3 survey areas in Lomami National Park and its buffer zone, Democratic Republic of the Congo. We used this method to evaluate the species richness, community composition, and vertical habitat use of non-volant mammals. We identified 47 mammal taxa, including 8 outside their documented geographic range. Species accumulation curves showed that pooling detections across strata increased estimated richness relative to single-stratum sampling. However, pairwise pooling of strata to produce rarefaction curves revealed that combining ground and canopy cameras can sufficiently estimate species richness, suggesting redundancy in understory detections. Ordination analyses and height-distribution data revealed strong vertical structuring of mammal communities, with understory detections overlapping both terrestrial and canopy assemblages and capturing transitional habitat use. Notably, one-quarter of the arboreal taxa favored the understory-midstory level, indicating that reduced-strata surveys may mischaracterize vertical habitat use and bias analyses informed by presence/absence data. Our results demonstrate that while ground-canopy camera combinations may approximate species richness, full vertical sampling is necessary to accurately interpret community structure and ecological roles of species. Multi-strata camera columns provide a scalable approach for biodiversity assessment and conservation monitoring in structurally complex tropical forests.

## Teaser

Using vertically aligned “camera columns” from forest floor to canopy in the Congo Basin, we recorded 47 mammals, including 8 range expansions. While ground-canopy sampling may be sufficient for inventories, full multi-strata surveys were essential to characterize the mammal community. 
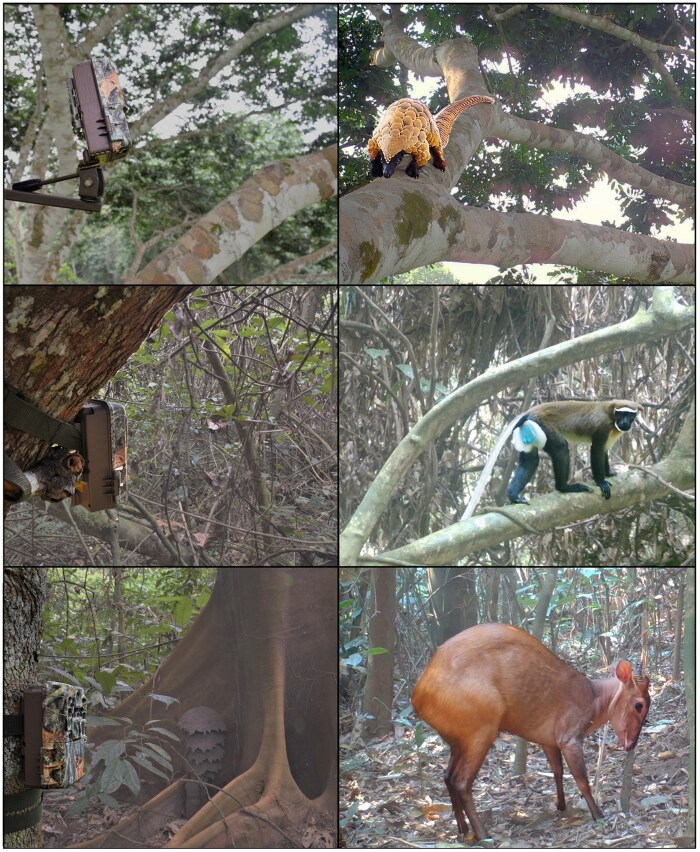

Human-driven biodiversity loss is a well-documented crisis, yet international targets aimed at reducing our impact on species and ecosystems are rarely met (Johnson et al. 2017). Given limited conservation funding, in situ efforts should maximize the benefits across multiple species (Plumptre et al. 2021) and doing so requires knowledge of the distribution and population status of species that are difficult to detect (Whittaker et al. 2005; Anthony et al. 2015; Bland et al. 2016). However, research on such species is often deprioritized because it requires intensive effort and risks yielding few results (Brooks et al. 2004). Despite the risks, such exploratory research can be impactful by revealing range expansions, lost species, and undescribed species (Mwenja 2007; Fauna & Flora International 2008; JH Hart et al. 2012; Allgas et al. 2014; Li et al. 2015; Nater et al. 2017; Costa-Araújo et al. 2019; Nguyen et al. 2019; Heritage et al. 2020; Haysom et al. 2021; Akampurira et al. 2024).

Tropical rainforests, the most biodiverse terrestrial ecosystems, are increasingly threatened by deforestation and hunting (Mittermeier et al. 1998; Ripple et al. 2016; Turubanova et al. 2018). These vertically stratified environments support complex ecological communities from the forest floor to the canopy (Wilson and Moffett 1991; Moffett 2000). More than half of all terrestrial vertebrate species live in rainforests, and approximately 75% of these are arboreal (Mittermeier et al. 1998; Kays and Allison 2001; Pillay et al. 2022). Arboreal mammals are key contributors to seed dispersal and nutrient cycling (Monteza-Moreno et al. 2022), but are especially vulnerable to anthropogenic disturbance (Dumbrell and Hill 2005; Klimes et al. 2012; Whitworth et al. 2019). Resource stratification (e.g., food and shelter) and structural heterogeneity create diverse niches across forest strata, shaping species distributions and interactions (Terborgh 1992; Grelle 2003; Lambert et al. 2005; Ferreira de Camargo et al. 2018; Basham et al. 2023). Accordingly, arboreal mammals may exhibit height-specific preferences reflecting differences in resource use, locomotor capabilities, and microclimate (Bowler et al. 2017; Whitworth et al. 2019; Laughlin et al. 2020; Godoy-Güinao et al. 2023). Despite their ecological importance, arboreal mammals are often underrepresented in biodiversity surveys (Ahumada et al. 2013; Rovero et al. 2014; Beaudrot et al. 2016).

Canopy research, described as the last biological frontier (due to the relatively limited study of this highly diverse ecosystem), has expanded since the 1980s and emphasizes the role of forest strata in ecological processes (Wilson and Moffett 1991; Ozanne et al. 2003). Camera traps (hereafter, cameras) are becoming an increasingly popular tool to study arboreal mammals (Moore et al. 2021). Some researchers have placed cameras in the understory to midstory to target specific species (Otani 2001; Kierulff et al. 2004; Schipper 2007; Oliveira-Santos et al. 2008; Dalloz et al. 2012; Olson et al. 2012; Tan et al. 2013; Alempijevic et al. 2021). Advances in canopy-access techniques have enabled researchers to deploy cameras at midstory and canopy levels, allowing a more complete inventory of arboreal mammal communities (Gregory et al. 2014; Rivas-Romero and Soto-Shoender 2015; Whitworth et al. 2016; Bowler et al. 2017; Haysom et al. 2021; Masseloux et al. 2023). Surveys that sample only a narrow vertical range may fail to characterize the mammal community, particularly in structurally complex forests (Malcolm 1991; Francis 1994; Tregidgo et al. 2010; Marques et al. 2016).

Several studies have demonstrated the value of sampling different combinations of forest strata to inventory or characterize mammal communities. Most of these studies have paired ground and arboreal cameras (Jayasekara et al. 2007; Gregory et al. 2017; Fang et al. 2018; Lama 2018; Whitworth et al. 2019; Tongkok et al. 2020; Moore et al. 2020; Arévalo-Sandi et al. 2021; Chen et al. 2021; Haysom et al. 2021; Mande et al. 2023). Cassano et al. (2012) compared mammal detections between the ground and understory (3–4 m). Akampurira et al. (2024) aligned 3 cameras vertically between the ground and midstory (∼0 m, 5 m, and 10 m). Hongo et al. (2020) and Godoy-Güinao et al. (2023) sampled across forest strata, but inconsistently between sampling sites.

The Congo Basin is one of the world’s most intact rainforests. The Democratic Republic of the Congo (DRC) holds 60% of this forest, but insecurity and limited infrastructure contribute to knowledge gaps in biodiversity data; and for many species, basic ecological information remains undocumented (Myers et al. 2000; Anthony et al. 2015; Breuer et al. 2016). Increasing trends in both primary forest loss and bushmeat hunting pose a significant threat to forest mammals (Wilkie and Carpenter 1999; Ripple et al. 2016; Turubanova et al. 2018; Global Forest Review 2024). Few arboreal camera surveys have been conducted in African forests (Moore and Niyigaba 2018; Hongo et al. 2020; Linden et al. 2020; Matsuda Goodwin et al. 2020; Moore et al. 2020; Bersacola et al. 2022; Koffi et al. 2022; Mande et al. 2023; African Parks 2024; Akampurira et al. 2024; Moore et al. 2024), and, to our knowledge, all prior surveys in the DRC have occurred on the forest floor (TB Hart et al. 2012; Sakamaki et al. 2016; Bessone et al. 2020; Houngbégnon et al. 2020; Fasbender et al. 2022; Fournier et al. 2023; Van Der Hoek et al. 2023; Van Vliet et al. 2023; Bessone et al. 2025; Balduccio et al. 2025) with the exception of Alempijevic et al. (2021, 2022), Alempijevic (2023, Chapter 4) and Mande et al. (2023).

Alempijevic et al. (2022) deployed multi-strata cameras in Lomami National Park (LNP) and the buffer zone to study the elusive Dryas Monkey (*Chlorocebus dryas*). Their systematic “camera column” method, with 3 vertically aligned cameras per sampling site—representing the entire vertical profile of the forest—produced valuable bycatch data revealing the broader mammal community. Alempijevic (2023) later deployed camera columns in a forest–savanna ecotone using an occupancy framework.

In this study, we evaluate the effectiveness of the camera column method using data collected by Alempijevic et al. (2022) and Alempijevic (2023) to characterize non-volant mammal communities in tropical forests. To highlight the contribution of each camera in the column, we compare species richness detected per stratum and by different combinations of strata. We reveal the vertical distribution of stratum use of each identified taxon, including small-bodied mammals that are often excluded from similar studies. We highlight findings that address knowledge gaps that could better inform IUCN Red list assessments and stimulate further research on this poorly studied mammal community. We also make practical considerations regarding the implementation of camera columns.

## Methods

### Ethics statement

This non-invasive research used infrared camera traps. We received research permissions from the Institut Congolais pour la Conservation de la Nature (ICCN) to access Lomami National Park and community leaders to access the buffer zone. Florida Atlantic University’s Institutional Review Board [916126-2] and Institutional Animal Care and Use Committee [A19-34] reviewed our protocols. This research also meets the 2016 Guidelines of the American Society of Mammalogists for the use of wild mammals in research and education (Sikes et al. 2016).

### Study areas

**Fig. 1:**
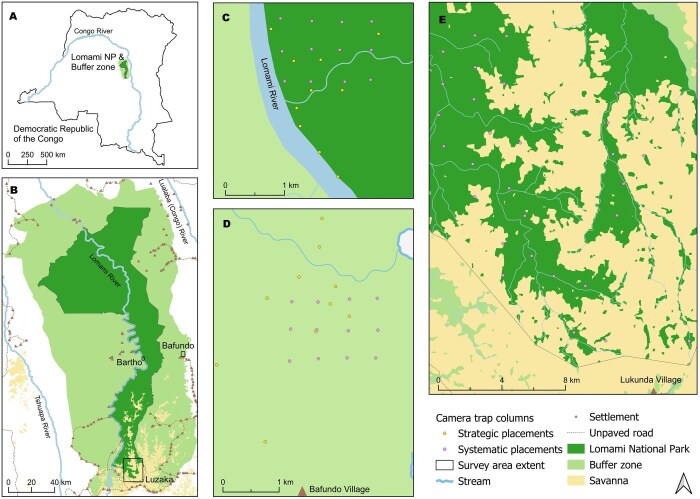
Lomami National Park and its buffer zone shown within the Democratic Republic of the Congo (A) and within the Tshuapa-Lomami-Lualaba river landscape (B). Continuous lowland rainforest covers the majority of the landscape and transitions to a forest-savanna ecotone in the south. Camera columns were deployed in sites within Bartho (C), Bafundo (D), and Luzaka (E) survey areas.

This study includes 3 camera surveys conducted within Lomami National Park (LNP) and its buffer zone in Maniema Province, DRC (Fig. 1). LNP spans a longitudinal gradient from equatorial rainforest to seasonally dry forest-savanna ecotone, conserving 8,879 km^2^ of wilderness. A 35,000 km^2^ buffer zone surrounds the park, supporting local communities dependent on natural resources. Although eco-guards conduct anti-poaching patrols and collect biodiversity data, these efforts tend to favor conspicuous species and underrepresent overall diversity (Burton 2012).

The 3 study areas are within the Lomami–Lualaba interfluve. Elevation ranges between 428 and 511 m asl. The majority of the study areas contain mixed-species terra firme forest, but all experience some seasonal inundation along watercourses. Zingiberales herbs and lianas dominate the understory and typically occur in low densities in closed-canopy forest while forming dense tangles in canopy gaps.

The Bafundo study area (2.089200° S, 25.575000° E) is in the LNP buffer zone, where a seasonal hunting ban is enforced from June 4 until September 30. This forest was inhabited by the Bafundo village between 1935 and 1956, after which the settlement relocated 3 km south. The resulting landscape is a mosaic of primary forest, secondary growth, and fallow fields. The understory was most dense at Bafundo and notably contained a high abundance of rattan palms. The Bartho study area (2.104620° S, 25.264310° E) includes an abandoned fishing camp (Camp Bartho) on the east bank of the Lomami River, approximately 30 km west of Bafundo. Compared to Bafundo, we observed minimal evidence of anthropogenic disturbance in Bartho. The Luzaka study area (2.988494° S, 25.170896° E) is 100 km south of Bartho in the park’s southern sector. Luzaka includes Marantaceae-rich gallery forests (Pouteau et al. 2024) and scattered tree islands amid a savanna dominated by tussock grasses and sedges on poorly draining white-sand soils. Luzaka experiences a dry season from June to September (Fasbender et al. 2022) and is subject to the most seasonal inundation among the 3 study sites.

### Camera column placement

**Fig. 2:**
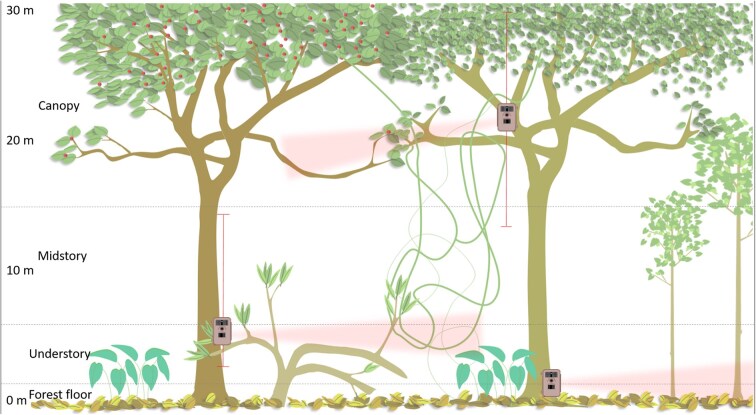
Schematic diagram of the camera column method. Cameras are placed facing parallel to the forest floor, understory-midstory, and canopy; forming a vertical column of mammal surveillance at a sampling site. Horizontal beams indicate each camera’s detection zone. Vertical bars indicate the range of heights each camera was placed throughout the surveys, which was dependent on the canopy height and forest structure at each sampling point.

We selected sampling sites based on study-specific objectives outlined below. We used a multi-strata “camera column” technique, systematically placing 3 cameras at each site: 1 each on the forest floor, in the understory-midstory, and in the canopy (Fig. 2). Upon arriving at each site, we selected appropriate camera positions in all 3 strata, which were dependent on site-specific canopy height and forest structure (See Supplementary Data SD1 for heights of each camera placed in this study). Camera positions were chosen based on the availability of substrate that could facilitate either arboreal or terrestrial locomotion, with no bait used nor other resources considered (i.e., tree species). Ground cameras were mounted 0.2–1.0 m above ground (¯$\underline{x\;}$± SD; 0.07 ± 0.75 m), typically along game trails, but occasionally on stream banks or in open areas. Understory-midstory cameras were placed at 1.5–14.2 m (5.11 ± 3.12 m), targeting dense vegetation such as liana tangles, shrubs, or horizontal branches. We favored the understory when structurally complex vegetation was present, and only placed cameras higher in the midstory at sites where understory structure was sparse. Hereafter, mention of understory cameras refers to placements in either the understory or midstory strata. Canopy cameras were mounted 12.3–29.0 m above the ground (20.21 ± 3.85 m), facing large horizontal limbs or branch clusters that connected neighboring crowns (Gregory et al. 2014). We accessed the canopy using the single-rope technique, and maneuvered laterally using the double-rope technique when needed (Maher 2010). After installing the canopy cameras, we left a braided nylon mason line to facilitate future access for camera maintenance and retrieval.

All cameras were set to record high-quality videos, allowing for both improved species identification and behavioral observations (Green et al. 2023). Most were angled slightly downward by placing a 7–10-mm branch segment behind the mounting strap. Where finer positioning was needed, we used HME trail camera mounts with 360-degree rotation. Cameras were operated continuously over 24 h periods and were set to high sensitivity to detect fast-moving animals in warm conditions (Gregory et al. 2014). We powered each camera with 8 Energizer lithium AA batteries and used 32 or 64 GB SanDisk SD cards. Depending on the accessibility and survey design, we revisited sites up to twice to ensure camera functionality. However, the cameras deployed in Bartho were not checked during the survey; some remained functional for up to 8 mo post-deployment.

Camera positions were verified in the field by triggering and reviewing the subsequent recordings to confirm the desired field of view, typically with the focal substrate in the lower third of the camera. We recorded metadata for each deployment, including the camera ID, column site, date, time, stratum, and height. To minimize errors during data logging and trap-effort calculations, we triggered each camera while reciting its relevant metadata before the team departed from the site and upon returning.

### Bafundo and Bartho: Dryas Monkey-targeted surveys (2016–2018)

We conducted a two-phase survey in Bafundo and Bartho to evaluate the habitat use by Dryas Monkeys (Alempijevic et al. 2022). In Phase I, we strategically deployed 10 camera columns at fallow field edges and structurally complex forest, using local knowledge. Informants led us to sites ranging between 400 m and 2.1 km apart, along footpaths. Phase II was a systematic survey, where we deployed 12 camera columns 500 m apart in a grid across the same areas to compare Dryas Monkey detection rates between strategic and systematic sampling. In total, we used 55 Bushnell Trophy Cam models (119636, 119736, 119774, and 119837). Cameras were programmed to record 60 s videos to increase the chances of recording as much phenotype and behavioral data on Dryas Monkeys as possible (Alempijevic et al. 2021).

### Luzaka: mammal occupancy survey (2021)

We designed the Luzaka survey to estimate the occupancy of mammal species across a forest–savanna ecotone (Alempijevic 2023; Gorczynski et al. in press). Using *ArcMap 10.7.1* (ESRI 2019), we selected thirty sampling sites 2 km apart spanning 172 km^2^ of gallery forest, ecotone, and isolated tree islands. The spacing met the assumption of independence between column sites for occupancy modeling (MacKenzie et al. 2006; Bowler et al. 2017). We deployed 30 camera columns, using 90 Browning Recon Force HD Advantage 20 MP cameras. Cameras were programmed to record 30 s videos; and the Smart IR Video setting available on this model allowed for longer recordings if animals remained active beyond this time limit.

### Video processing

For the Bafundo and Bartho surveys, we reviewed all videos using Mammals of Africa (Kingdon 2015) as a reference, and we sought expert opinion when needed. Each detection was logged with the date, time, camera ID, taxon (to the lowest possible level), and observed behavior. To differentiate morphologically similar arboreal rodents, we compared field images (Supplementary Data SD2) with specimens at the American Museum of Natural History (Supplementary Data SD3). We excluded bats, shrews, and small murids (mice and rats) from the analyses due to identification uncertainty, but included all mammals identifiable to the genus. For congeners identifiable to species only in some instances (e.g., via vocalizations or high-quality footage), we aggregated records at the genus level for analyses (Funisciurus and Galagoides). However, we included all verified species in our total count.

To manage the Luzaka dataset, we trained volunteers using validated data and assigned them batches of videos for processing. After completing their batch, volunteers re-reviewed the batch of one another as secondary observers. Discrepancies were resolved and spot-checked for quality assurance.

Species detections were grouped into a single “event” when they occurred within 30 min of the same species appearing at the same camera (Laughlin et al. 2020; Haysom et al. 2021). Trap-days were calculated per stratum as the number of days a functional camera was active. If a camera failed by the time of retrieval, we used the last recorded video as the endpoint.

### Species accumulation and richness estimation

To compare species richness among strata and survey areas while accounting for unequal sampling effort, we generated sample-based rarefaction curves using the “vegan” package in R (Oksanen et al. 2025). Species events were aggregated by camera trap-day to standardize sampling effort. To assess if sampling across all 3 strata is necessary to estimate mammal richness, we generated additional curves from each pairwise combination of strata (ground-understory, canopy-understory, and canopy-ground) and all strata pooled. To estimate total species richness within each survey area, we used non-parametric richness estimators using the “specpool” function in “vegan” (Oksanen et al. 2025). We report Bootstrap, Chao2, and first-order Jackknife (Jackknife 1) estimates with associated standard errors. These estimators infer asymptotic richness from the frequency of rare species based on incidence pooled across all samples collected in a survey area.

### Vertical stratum distribution

We used three-dimensional non-metric multidimensional scaling (NMDS) to visualize patterns in mammal community composition among camera column sites. NMDS is well suited for ecological community data because it is based on ranked dissimilarities and does not assume linear relationships or multivariate normality. We calculated dissimilarities using the Bray-Curtis index, which is robust to joint absences and emphasizes differences in relative abundance among sites (Bray and Curtis 1957; Clarke 1993; Legendre and Legendre 2012) in “vegan.” To visualize the breadth of stratum use for each identified mammal, we plotted mammal events across the range of camera heights using “ggplot2” (Wickham 2016), combining data from all surveys. Boxplots were generated for each mammalian order with arboreal representatives; pangolins and afrotherians were combined for visualization.

### Effort and time requirements for camera column setup

We set an average of 2 camera columns per day, regardless of the inter-site distance. Depending on site accessibility, it took a team consisting of 1 climber and 2 to 5 assistants between 1 and 3 wk to complete the setup in each survey area. For maintenance and retrieval, the team completed an average of 3 columns per day. Canopy camera placement required between 1 and 5 h per site and was completed by the lead author. Camera placements in the other strata typically required 5–20 min each and were usually installed concurrently by team members during canopy setup.

## Results

### Descriptive results

The survey duration, site selection, number of camera columns deployed, total trap-days, and accumulated mammal detection events varied across the 3 study areas (Table 1). In total, we identified 47 mammal taxa at the genus or species level in the orders Primates (11 species; Supplementary Data SD4), Carnivora (11; Supplementary Data SD5), Rodentia (11; Supplementary Data SD6), Artiodactyla (9; Supplementary Data SD7), Pholidota (3; Supplementary Data SD8), and in superorder Afrotheria (2; Supplementary Data SD9). Cameras placed on the forest floor detected 32 taxa, including 9 arboreal species (*Cercopithecus wolfi*, *C. neglectus*, *C. ascanius*, *Galagoides* sp., *Nandinia binotata*, *Graphiurus* sp., *Heliosciurus rufobrachium*, *Funisciurus* sp., *Phataginus tricuspis*). Understory cameras recorded 23 taxa, and canopy cameras recorded 22 taxa (See Supplementary Data SD10 for detection frequencies and naïve occupancy rates per survey and stratum). Twenty-five percent of the identified mammals (50% arboreal) were classified as Near Threatened, Vulnerable, or Endangered according to the IUCN Red List. Eight species were detected outside of their documented range (Based on current IUCN Red List assessments; Table 2).

**Table 1:** Summary of multi-strata camera column surveys completed in the central and southern sectors of Lomami National Park and its buffer zone.

Survey area	Survey duration	Site selection	Camera columns	Trap days/stratumGround Understory Canopy			Mammal events
Bafundo Forest (Dryas-targeted)	Oct-Nov 2016	Strategic Local knowledge	10	269	299	294	639
	Sep 2017–Mar 2018	Systematic (500 m)	12	420	586	1156	1306
Camp Bartho (Dryas-targeted)	Nov 2016–Jul 2017	Strategic Local knowledge	10	700	1582	1143	1773
	Aug-Sep 2017	Systematic (500 m)	12	353	354	334	811
Luzaka (Occupancy)	Jun-Dec 2021	Systematic (2 km)	30	2629	2569	2416	3218

Bafundo and Bartho surveys were a study of Dryas Monkeys in continuous lowland forest of contrasting anthropogenic disturbance, while the Luzaka survey was a mammal occupancy study in a seasonally dry, forest gallery-savanna ecotone.

**Table 2:** The 8 mammal species detected during camera column surveys at Lomami National Park sites Bartho (BAR) and Luzaka (LUZ), and at the buffer zone site Bafundo (BAF), that were detected outside of their documented ranges based on the IUCN Red list.

Species	Survey areas	Notes	References
*Chlorocebus dryas*	BAF BAR LUZ	400 km range expansion documented. Rare observations in gallery forest are likely dispersing males.	(Hart et al. 2019; Alempijevic et al. 2022; Alempijevic 2023)
*Perodicticus* spp.	BAF BAR LUZ	Undocumented between Lomami-Lualaba interfluve. Specimen required to determine species.	(de Jong et al. 2019; Svensson and Pimley 2019)
*Lupulella (Canis) adustus*	LUZ	In Maniema, undocumented north of Kibombo. Range extends >100 km north through the savanna ecotone region of southern LNP.	(Hoffmann 2014)
*Petrodromus tetradactyla*	BAF BAR LUZ	Within the Lomami-Lualaba interfluve, undocumented outside of Kisangani ecoregion	(Rathbun and FitzGibbon 2015)
*Heliosciurus rufobrachium*	BAF BAR LUZ	Undocumented south of the Congo River outside of Kisangani ecoregion	(Cassola 2016a; Baelo et al. 2018)
*Protoxerus stangeri*	BAF BAR LUZ	Undocumented south of the Congo River outside of Kisangani ecoregion	(Cassola 2016b; Baelo et al. 2018)
*Paraxerus boehmi*	BAF BAR LUZ	Undocumented south of the Congo River outside of Kisangani ecoregion	(Cassola 2016c; Baelo et al. 2018)
*Idiurus* spp.	BAF BAR LUZ	Undocumented south of the Congo River. Specimen required to determine species.	(Hutterer 2016; Hutterer and Decher 2016)

### Species accumulation and richness estimation

**Fig. 3:**
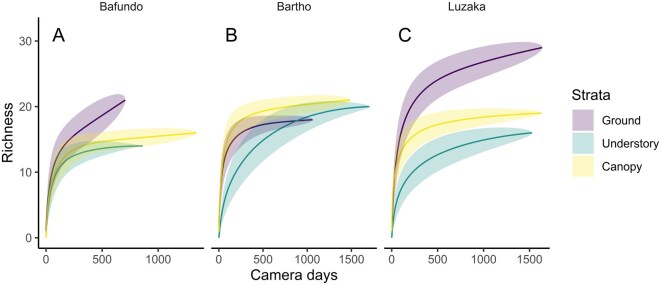
Sample-based rarefaction curves for mammal species richness by vertical stratum in (A) Bafundo, (B) Bartho, and (C) Luzaka survey areas. Ground, understory, and canopy strata are shown separately within each survey area. Shaded ribbons indicate ± 2 SD from rarefaction resampling.

**Fig. 4:**
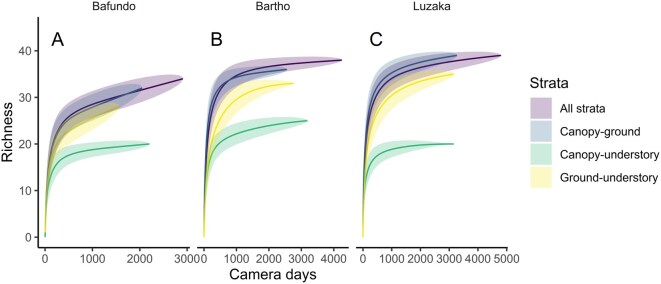
Sample-based rarefaction curves showing species accumulation for all strata pooled and all pairwise strata combinations in (A) Bafundo, (B) Bartho, and (C) Luzaka survey areas. Shaded ribbons indicate ±2 SD from rarefaction resampling.

**Fig. 5:**
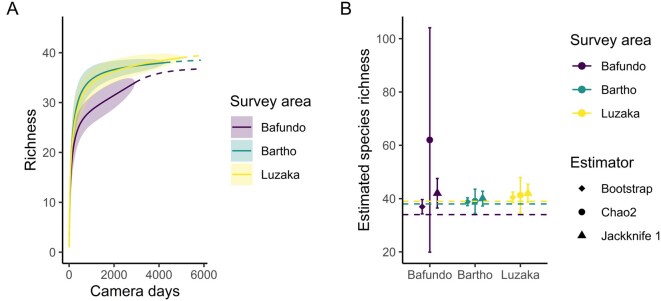
(A) Sample-based rarefaction curves for all-strata pooled within each survey area. Solid lines represent observed rarefaction curves, while dashed segments represent extrapolation toward the Bootstrap richness estimate. Shaded ribbons indicate ± 2 SD from rarefaction resampling. (B) Nonparametric species richness estimates from all-strata pooled species data from each survey area. Points represent Bootstrap, Chao2, and Jackknife 1 estimates; error bars indicate 95% confidence intervals. Horizontal dashed lines denote observed richness for each survey area.

Rarefied species accumulation curves reveal patterns in species accumulation and estimated richness among vertical forest strata and survey areas. When comparing strata individually across survey areas, cameras at ground-level accumulated the most species in Bafundo and Luzaka, while canopy-level cameras accumulated the most species in Bartho (Fig. 3). When we compared paired strata combinations and all strata pooled, the canopy-ground camera combination achieved similar results as the cameras pooled across all 3 strata (Fig. 4). Observed pooled richness was 34, 38, and 39 mammal taxa in Bafundo, Bartho, and Luzaka, respectively (Fig. 5). Bootstrapped estimates of species richness were slightly higher than observed values: Bafundo = 36.96 (CI = 34.28–39.64); Bartho = 38.83 (37.37–40.30); Luzaka = 40.48 (38.49–42.47). Jackknife 1 estimates were similar (Bafundo = 42.00; Bartho = 40.00; Luzaka = 42.00). Chao 2 also approximated species richness for Bartho (39.00) and Luzaka (41.25); however, it produced a substantially higher estimate for Bafundo with wide confidence intervals (61.99, 19.90–104.08).

### Vertical stratum distribution

**Fig. 6:**
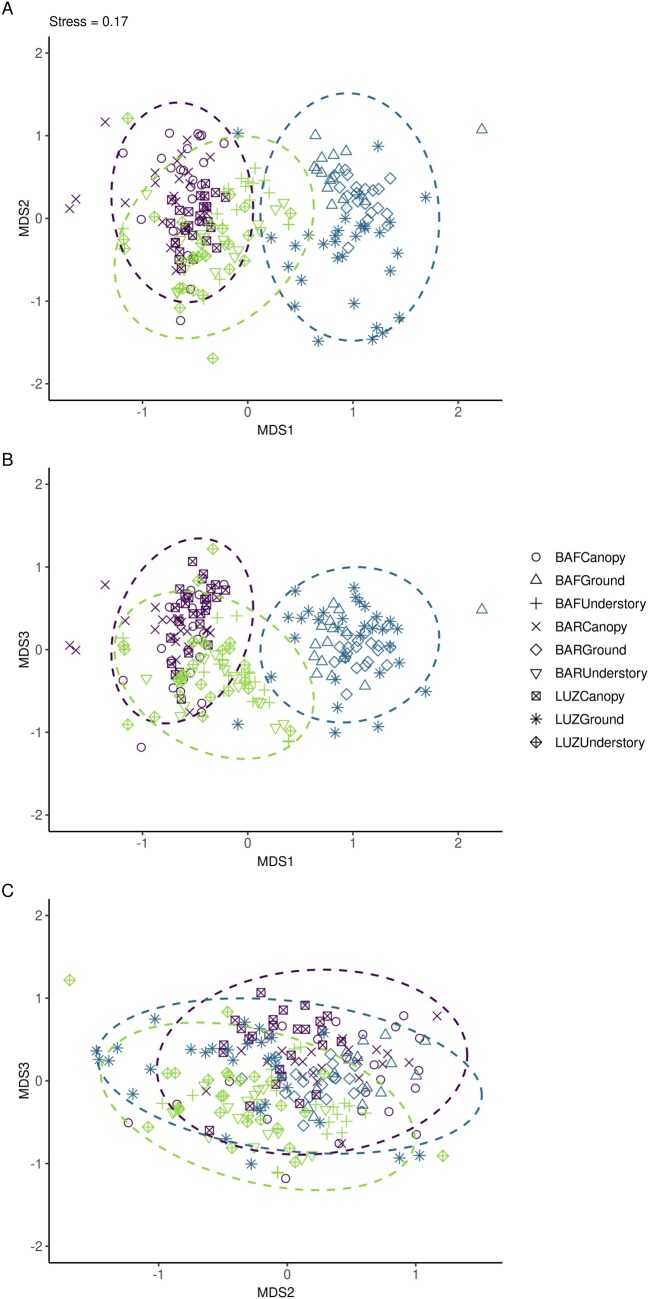
Three-dimensional non-metric multidimensional scaling plot for mammal communities detected at cameras set in 3 strata: ground (blue), understory (green), and canopy (purple) across 3 survey areas. Points represent individual cameras, and symbology represents the site and strata in which each camera was deployed. Each panel (A–C) shows a different pairwise comparison between the 3 NMDS axes.

Three-dimensional NMDS ordination revealed distinctive clustering of species composition by stratum (Fig. 6). The ordination had a stress value of 0.17, indicating an acceptable representation of community dissimilarities (stress <0.2). Ground and canopy cameras formed separate clusters, whereas understory cameras occupied an intermediate, overlapping space. The ordination suggested that species composition was more strongly structured by forest strata than by survey area.

**Fig. 7:**
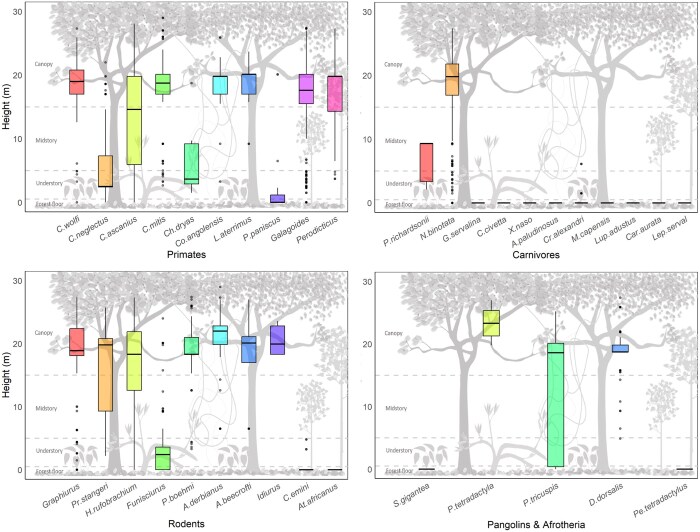
The height above ground at which mammals were detected by cameras during camera column surveys in Lomami National Park and buffer zone. Boxes represent the median height with upper and lower quartiles where each species was detected (25% greater and 25% lesser than the median); whiskers represent maximum/minimum values, and dots outliers. Ungulates are completely terrestrial, thus are not shown.

Stratum use varied markedly across and within taxonomic orders (Fig. 7). Primates, rodents, and pangolins exhibited the highest degree of arboreality; whereas carnivores and ungulates were primarily terrestrial. We detected the only known arboreal afrotherian (*Dendrohyrax dorsalis*) in the region, but only 1 of the 4 terrestrial species (*Petrodromus tetradactylus*). Seventeen mammal taxa were most often detected in the canopy; 5 (excluding *Galagoides demidoff* and *Funisciurus congicus*) in the understory; and 23 on the forest floor.

## Discussion

### Effectiveness of the camera column method

The camera column method proved to be highly effective for characterizing the non-volant mammal community in our study areas. We inventoried a relatively high number of species, which we attribute to our multi-strata survey design and our use of video rather than still images, which allowed us to detect subtle morphological and behavioral cues that aided in distinguishing similar species. Camera columns did fail to detect 1 large mammal which we observed during fieldwork in Luzaka, the African Forest Buffalo (*Syncerus caffer nanus*). Other undetected mammals presumably occur at low densities or outside of the study areas, and may require targeted camera placements. Surveying small nocturnal mammals such as bats, shrews, and murids is best achieved using other techniques such as acoustic monitoring, live trapping, or eDNA sampling (Taylor et al. 2018; Broadhurst et al. 2025). Nonetheless, camera columns documented a Congo Basin mammal community and provided ecological insights that would not have been possible using single-stratum or paired-strata surveys.

### Species accumulation and estimated species richness

Our results confirm that aggregating detections across strata increases estimates of species richness, which is consistent with previous multi-strata studies (Ferreira de Camargo et al. 2018). However, pairwise pooling of strata in rarefaction curves showed that combining canopy and ground cameras recovered species richness comparable to that obtained when all strata were pooled, whereas pooling canopy and understory cameras, or understory and ground cameras did not (Fig. 4). Therefore, to approximate mammal richness in tropical forests, paired canopy-ground sampling may be sufficient.

Nonparametric estimators of species richness generally converged on observed richness at Bartho and Luzaka. However, Chao2 produced an elevated and uncertain estimate at Bafundo (Fig. 5). Chao2 is sensitive to the ratio of singletons to doubletons in the incidence matrix (Chao 1989; Colwell and Coddington 1994) and Bafundo had a relatively high prevalence of species that were only observed on 1 camera trap-day. Similarities between the Bootstrap and Jackknife estimates, which are less sensitive to the influence of rarely detected species, suggest that true richness at Bafundo is unlikely to approach the upper bound implied by Chao2. The relatively high number of singleton species detections and low frequency of doubletons in Bafundo may reflect lower abundance due to hunting pressure in the buffer zone. Indeed, most singletons at Bafundo were mammals targeted by hunters that were detected more frequently in the other survey areas.

Rarefaction curves revealed broad patterns in species richness across survey areas. Bafundo exhibited the lowest estimated richness, likely caused by anthropogenic disturbance, while Bartho supported the highest arboreal richness. Two primates (*C. mitis* and *Lophocebus aterrimus*) were frequent in Bartho but rare or absent in Bafundo and Luzaka, likely reflecting overhunting in Bafundo and natural absence in Luzaka. Conversely, Frogge et al. 2022 found that the *C. mitis* population in Kibale National Park was limited by competition with *L. albigena*. Fasbender (unpublished report 2022) hypothesized that the rarity of Fabaceae and Ebenaceae trees in Luzaka could explain the apparent absence of both *C. mitis* and *L. aterrimus* in that area.

In contrast, Luzaka exhibited the highest terrestrial richness, likely due to extensive coverage of forest–savanna ecotones and broad spatial sampling, which increased detection of both forest- and savanna-adapted species. Two savanna carnivores (*Lupulella adusta* and *Leptailurus serval*) were detected at forest-savanna ecotones along with forest-dwelling species in Luzaka. Admittedly, the Bafundo and Bartho study areas were designed for comparison, and the Luzaka study differed spatially and temporally; thus, comparisons should be considered cautiously. Specifically, the former were designed to investigate observed affinity of the Dryas Monkey for disturbed forest, and sampled a relatively small area of forest, while the latter had lower sampling intensity of a larger and more heterogeneous landscape.

Forest structure may have also influenced richness patterns. Luzaka and Bafundo survey areas had more edge habitat, driven by aforementioned natural and anthropogenic factors, conditions that encourage arboreal mammals to descend and increasing detectability at lower strata (Malcolm 1991; Struhsaker 1997; Malcolm and Ray 2000; Ferreira de Camargo et al. 2018). Indeed, *N. binotata*, *H. rufobrachium*, and *P. tricuspis* were most frequently detected at lower heights in Bafundo and more arboreal species were detected at ground-level in Luzaka than the other survey areas (Supplementary Data SD10). Interestingly, while ground-canopy camera studies (Gregory et al. 2014; Whitworth et al. 2016, 2019; Haysom et al. 2021) found that additional effort was needed in the canopy, our results aligned with Hongo et al. (2020) who concluded that additional effort was needed on the ground.

### Vertical stratification

Canopy-ground sampling may be sufficient to inventory the majority of the mammal community in tropical forests (Cassano et al. 2012; Gregory et al. 2017; Fang et al. 2018; Moore et al. 2020; Arévalo-Sandi et al. 2021), however, additional analyses revealed that reduced-strata surveys may obscure important aspects of vertical habitat use and community structure. The NMDS ordination plotted understory camera placements in an intermediate and overlapping space between canopy and ground samples, which clustered more distinctly. While this result also suggests that the canopy and ground assemblages are most important, excluding understory cameras oversimplifies community structure and masks vertical connectivity among strata. Indeed, some patterns in mammal community responses to environmental variables are only revealed when all forest strata are considered at each sampling point (Gorczynski et al. in press).

The significance of the understory level cameras is best visualized by examining the height distribution boxplot for each mammal (Fig. 5). Approximately one-quarter of the arboreal mammal community favored the understory. This includes species that we analyzed by genus. *F. anerythrus* and *F. congicus* both occupied the understory, while *G. thomasi* used the canopy and *G. demidoff* used the understory. The distribution of *Galagoides* detections (Fig. 5) likely reflects this difference in stratum use, with canopy records dominated by *G. thomasi* and understory outliers probably representing rare detections of *G. demidoff* occupying canopy gaps.

Many arboreal mammals occupied narrow ranges within the vertical columns. Without sampling both canopy and understory strata, patterns of stratum use would be oversimplified or go undetected. Arboreal species with restricted stratum use might be falsely characterized as rare, or missed entirely. Such bias may explain why Boehm’s Bush Squirrel (*Paraxerus boehmi*) was most often detected in the canopy, despite being associated with the understory in the literature (Cassola 2016c), or why *Poiana richardsonii* has previously been considered a canopy specialist (Van Rompaey and Colyn 2013). Our results align with Akampurira et al. (2024) and Moore and Niyigaba (2018) who report *P. richardsonii* at heights of 6–10 m.

Alternatively, shifts in vertical space use may reflect responses to forest structure or niche partitioning among similar species. *Poiana richardsonii* may be using lower heights to reduce competition with *N. binotata*, the species with the highest naïve occupancy in this study (Supplementary Data SD10); and perhaps *P. boehmi* utilizes higher strata in our survey areas to avoid competition with *Funisciurus*. Vertical stratification is a mechanism by which arboreal mammals may reduce interspecific competition, and is well documented in African primates (Gautier-Hion 1988; Cords 1990; McGraw 1994, 2004).

As expected, *Cercopithecus* monkeys showed strong vertical stratification: *C. neglectus* utilized the understory, *C. wolfi* and *C. mitis* the canopy, and *C. ascanius* exhibited the broadest vertical range, with frequent detections in the midstory. Camera column surveys are needed on the west bank of the Lomami River to better characterize the vertical habitat use of *C. lomamiensis*, which was among the most frequently detected terrestrial mammals in ground surveys (Fournier et al. 2023). The equally speciose, congeneric, and frugivorous *Cephalophus* duikers might be restricted to the forest floor, but forage on fruits of different sizes, partition temporally, and spatially (Houngbégnon et al. 2020). Remarkably, all 3 African forest pangolin species were detected in the Luzaka survey: *Phataginus tetradactyla* in the canopy, *Smutsia gigantea* on the ground, and *P. tricuspis* across all strata. *Phataginus tricuspis* exhibited the widest vertical range of any species in our study, and *P. tetradactyla*, the highest. To the best of our knowledge, this is the first study to document 3 sympatric pangolin species, and builds upon similar efforts to develop detection methods for these highly trafficked and poorly known mammals (Moore et al. 2024).

### Conservation and monitoring implications

From a conservation perspective, mischaracterizing vertical habitat use has important consequences. Species that may be relatively common but vertically constrained may be underestimated or overlooked in monitoring programs that rely on reduced-strata surveys, potentially biasing assessments of population status and habitat requirements. The Dryas Monkey is the epitome of this problem, being undetected in the Lomami Basin for years before it was determined to be restricted to the understory of disturbed forest, and has since been downgraded from Critically Endangered to Endangered (Hart et al. 2019). Of the threatened mammals that we detected, the camera column method is particularly useful for monitoring *P. tricuspis*, which had moderate to high naïve occupancy rates in all study areas. Surprisingly, *P. tricuspis* was detected most frequently at ground-level and at more sites at Bafundo, where it is at most risk of detection by hunters among the survey areas (Supplementary Data SD10). This pattern may reflect prey availability, as wood and litter-consuming termites are positively associated with disturbance in African forests (Eggleton et al. 2002).

By capturing most non-volant mammals occupying vertical forest strata, camera columns provide a more realistic representation of mammal communities and improve the ability to detect changes in community composition or behavior resulting from habitat degradation and unregulated hunting. Of the 8 species that have not been formally documented in the region, 6 were widespread. These records can inform range reassessments and call attention to the need to conduct similar research elsewhere in the Congo Basin. Of particular interest are the species that could not be identified. *Idiurus* was detected frequently in Bafundo, and has 2 recognized species (Supplementary Data SD11) north of the Congo River. *Perodicticus* was detected most frequently in Luzaka. *Perodicticus edwardsi* occurs just West of the Lomami River, and *P. ibeanus* East of the Lualaba, but genetic or morphological analysis is required to verify the species occurring in the Lomami-Lualaba interfluve (de Jong et al. 2019; Svensson and Pimley 2019). Relatively large and intact areas of forest persist in DRC, and exploratory field surveys are required to determine their conservation value (Fonteyn et al. 2023).

### Effort and practical considerations

Accessing the canopy was the most time and training intensive component of deploying the camera column surveys. The time to secure a safe canopy anchor point varied substantially according to forest structure. Closed-canopy forests with open understories were the most efficient for canopy access, whereas open-canopy or liana-rich areas presented greater challenges. Lambert et al. (2005) found that canopy trapping was less necessary in low-canopy or disturbed forests where most arboreal mammals were captured at understory levels. Similarly, arboreal mammals that prefer lower strata may not be worth surveying for in even-aged, closed canopy forest stands with simple understories. In such cases, preliminary surveys may help determine whether multi-strata sampling is warranted, or two-strata sampling is sufficient. However, while planning and fundraising for a canopy survey in structurally complex tropical forests, the marginal time and cost of adding ground and understory cameras should be considered, as they significantly enhance the sampling breadth. Furthermore, using this method in high canopy rainforests (≥40 m), may require additional cameras within the vertical column to capture the range of vertical stratum use by mammals (See Haysom et al. 2021, who set arboreal cameras at a range of 9.8–52.3 m).

### Notes on unidentified mammals and birds

Small nocturnal mammals constitute a significant component of the mammal community; however, cameras are not well suited to record most species. Unidentified murids accounted for 7.16% of all mammal events. Cameras captured murids in all strata, but mostly on the ground. A recent molecular analysis in Gabon identified 4 terrestrial and 1 arboreal murid (Martinez et al. 2025), suggesting that similar partitioning may have occurred among unidentified murid species in our study. Cameras rarely captured shrews on the ground or understory (0.39% of all mammal events). Bats comprised 2.01% of mammal detections and were recorded across all strata, exhibiting behaviors such as investigating fruit ripeness in the canopy. Notably, we recorded 16 events of microchiroptera repeatedly approaching other mammals (*C. dorsalis*, *P. tricuspis*, *N. binotata*; Supplementary Data SD12). In some videos, swarms of small insects, presumably parasitic dipterans, were seen pursuing mammals. Therefore, it is likely that these bats prey on flies attracted to other mammals. Similar behavior has been documented in North America (Palmer et al. 2019), suggesting a possible mutualism between microchiropterans and fly-infested mammals that warrants further study.

While we chose to focus on mammals, our study also yielded rich avian data. We recorded 491, 152, and 868 bird events on the ground, understory, and canopy cameras, respectively. Preliminary identification suggests that at least 17 avian orders were captured, but requires further investigation. We observed both predation (e.g., raptors feeding on mammals) and mixed-species interactions (e.g., *Horizocerus cassini* following monkey groups), reinforcing the utility of camera columns in broader ecological studies.

In conclusion, all species in a community are interconnected through their ecological interactions, and therefore declines in species abundance and extirpation reverberate through ecosystems (Johnson et al. 2017). Therefore, comprehensive surveys that include all forest strata are essential for understanding and conserving these systems. Our findings provide critical insights into mammal community composition and vertical stratification in the understudied Lomami Basin. While ground cameras detected 75% of mammal richness, little inference can be made about most arboreal species without cameras placed at their preferred heights. Camera columns offer an efficient and scalable approach for detecting rare species, evaluating conservation status, and guiding ecological research in tropical forests.

## Supplementary Material

gyag047_Supplementary_Data

## Data Availability

The data supporting the results of this study are archived at the Primatology Lab at Florida Atlantic University. Due to conservation sensitivity and permitting restrictions, the data are not publicly available but may be made available from the corresponding author upon reasonable request.

## Supplementary data

**Supplementary Data SD1.** Heights at which camera traps were placed for each column site in this study.

**Supplementary Data SD2.** *Funisciurus* and *Paraxerus* still frames from the same camera trap overlaid for comparison. Images are presented at the same scale to facilitate comparison of size, pattern, and body proportions among the 3 species.

**Supplementary Data SD3.** *Funisciurus* and *Paraxerus* skins for comparison to camera identifications. *Funisciurus congicus* and *F.c.interior* are shown (separated by a red dashed line) to exhibit variation in pelage between populations in drier regions outside of the Congo basin and populations within the basin. Left to right: *Funisciurus congicus* (AMNH# 86486); *F. c. interior* (AMNH# 2038); *F. anerythrus* (AMNH# 50835); *Paraxerus boehmi* (AMNH# 50674). Source: American Museum of Natural History.

**Supplementary Data SD4.** The 11 primates identified during camera column surveys in Lomami National Park and buffer zone. From Top Left to Bottom Right: *Cercopithecus wolfi*  ^NT^; *C. neglectus*  ^LC^; *C. ascanius*  ^LC^; *C. mitis*  ^LC^; *Chlorocebus dryas*  ^EN^; *Colobus angolensis*  ^VU^; *Lophocebus aterrimus*  ^VU^; *Pan paniscus*  ^EN^; *Galagoides thomasi**  ^LC^; *G. demidoff* * ^LC^; *Perodicticus* sp. ^LC^

*Confirmed by vocalization

**Supplementary Data SD5.** The 11 carnivores identified during camera column surveys in Lomami National Park and buffer zone. From Top Left to Bottom Right: *Poiana richardsonii ^LC^*; *Nandinia binotata ^LC^*; *Genetta servalina*  ^LC^; *Civettictis civetta*  ^LC^; *Xenogale naso*  ^LC^; *Atilax paludinosus*  ^LC^; *Crossarchus alexandri*  ^LC^; *Mellivora capensis*  ^LC^; *Lupulella adustus*  ^LC^; *Caracal aurata*  ^VU^; *Leptailurus serval*  ^LC^

**Supplementary Data SD6.** The 11 rodents identified during camera column surveys in Lomami National Park and buffer zone. From Top Left to Bottom Right: *Graphiurus* sp. ^LC^; *Protoxerus stangeri*  ^LC^; *Heliosciurus rufobrachium*  ^LC^; *Funisciurus congicus*  ^LC^; *F. anerythrus*  ^LC^; *Paraxerus boehmi*  ^LC^; *Anomalurus derbianus*  ^LC^; *A. beecrofti*  ^LC^; *Idiurus* sp. ^LC^; *Cricetomys emini*  ^LC^; *Atherurus africanus*  ^LC^

**Supplementary Data SD7.** The nine ungulates identified during camera column surveys in Lomami National Park and buffer zone. From Top Left to Bottom Right: *Philantomba monticola*  ^LC^; *Cephalophus nigrifrons*  ^LC^; *C. weynsi*  ^LC^; *C. silvicultor*  ^NT^; *C. dorsalis*  ^NT^; *Hyemoschus aquaticus*  ^LC^; *Tragelaphus spekii*  ^LC^; *T. eurycerus*  ^NT^; *Potamochoerus porcus*  ^LC^

**Supplementary Data SD8.** The 3 pangolins identified during camera column surveys in Lomami National Park and buffer zone. From Left to Right: *Smutsia gigantea*  ^EN^; *Phataginus tetradactyla*  ^VU^; *P. tricuspis*  ^EN^

**Supplementary Data SD9.** The 2 afrotherians identified during camera column surveys in Lomami National Park and buffer zone. Left: *Dendrohyrax dorsalis*  ^LC^; Right: *Petrodromus tetradactylus*  ^LC^

**Supplementary Data SD10.** Detection frequencies (Df) and naïve occupancy (naïve ψ) are reported for mammals detected in Lomami National Park and buffer zone. Df were calculated as the number of independent detection events per species recorded on the ground (G), understory (U), and canopy (C), standardized by trap effort, and expressed as events per 100 trap-days. Naïve ψ represents the percentage of camera columns within each study area at which a species was detected at least once during a survey, irrespective of vertical stratum, and without accounting for imperfect detection. For descriptive comparison, non-zero values were grouped into low, medium, and high (light-dark blue) categories based on natural breaks (Jenks optimization). Df; low (0.03–1), medium (>1–5), high (>5): naïve ψ; low (3.3–20%), medium (>20–50%), high (>50%). Zero values indicate non-detections and were not assigned categories. Species are ordered from highest to lowest total naïve ψ (sum of site-level values across Bafundo, Bartho, and Luzaka).

**Supplementary Data SD11.** *Idiurus* and *Graphiurus* skins for comparison to camera trap identifications. *Idiurus* and *Graphiurus* could not be identified to species from camera videos. Left to right: *Idiurus macrotis* (AMNH# 241152); *I. zenkeri* (AMNH# 50535); *Graphiurus christyi* (AMNH# 49893). Source: American Museum of Natural History.

**Supplementary Data SD12.** An unidentified microchiropteran repeatedly circles a white-bellied pangolin *Phataginus tricuspis* in the understory of the Bafundo survey area.
