# Supplementary figures and images for: Multi-strata “camera columns”: an effective approach to characterize non-volant mammal communities in tropical forests
Source: J Mammal. 2026 May 29;107(4):794–809. doi: 10.1093/jmammal/gyag047 (PMC13416187; doi:10.1093/jmammal/gyag047)

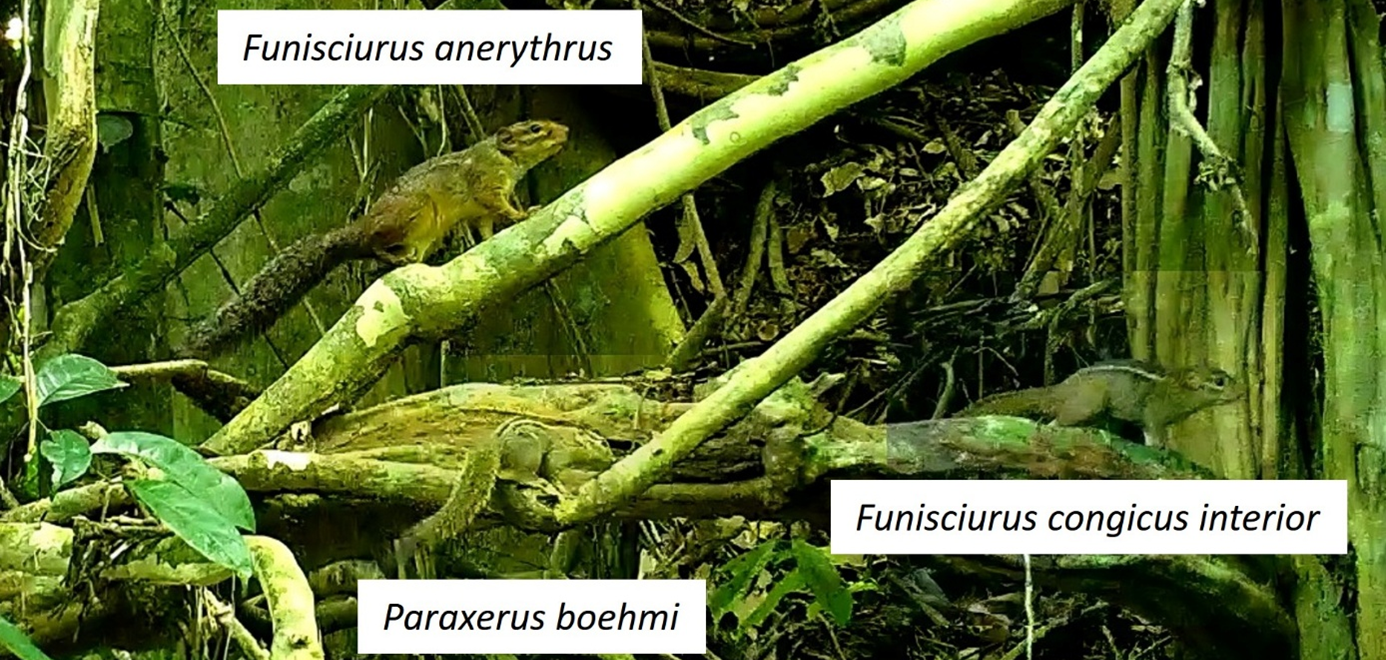

Supplement: gyag047_Supplementary_Data [file gyag047_supplementary_data.zip › Supplementary Data SD2.tif]

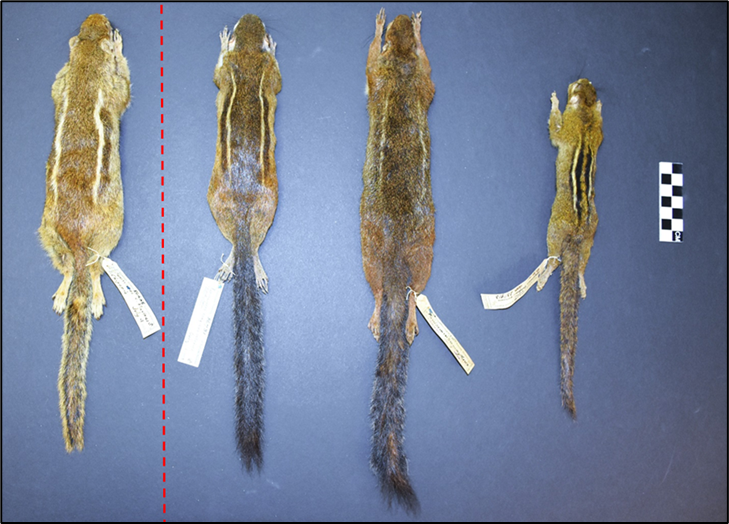

Supplement: gyag047_Supplementary_Data [file gyag047_supplementary_data.zip › Supplementary Data SD3.tif]

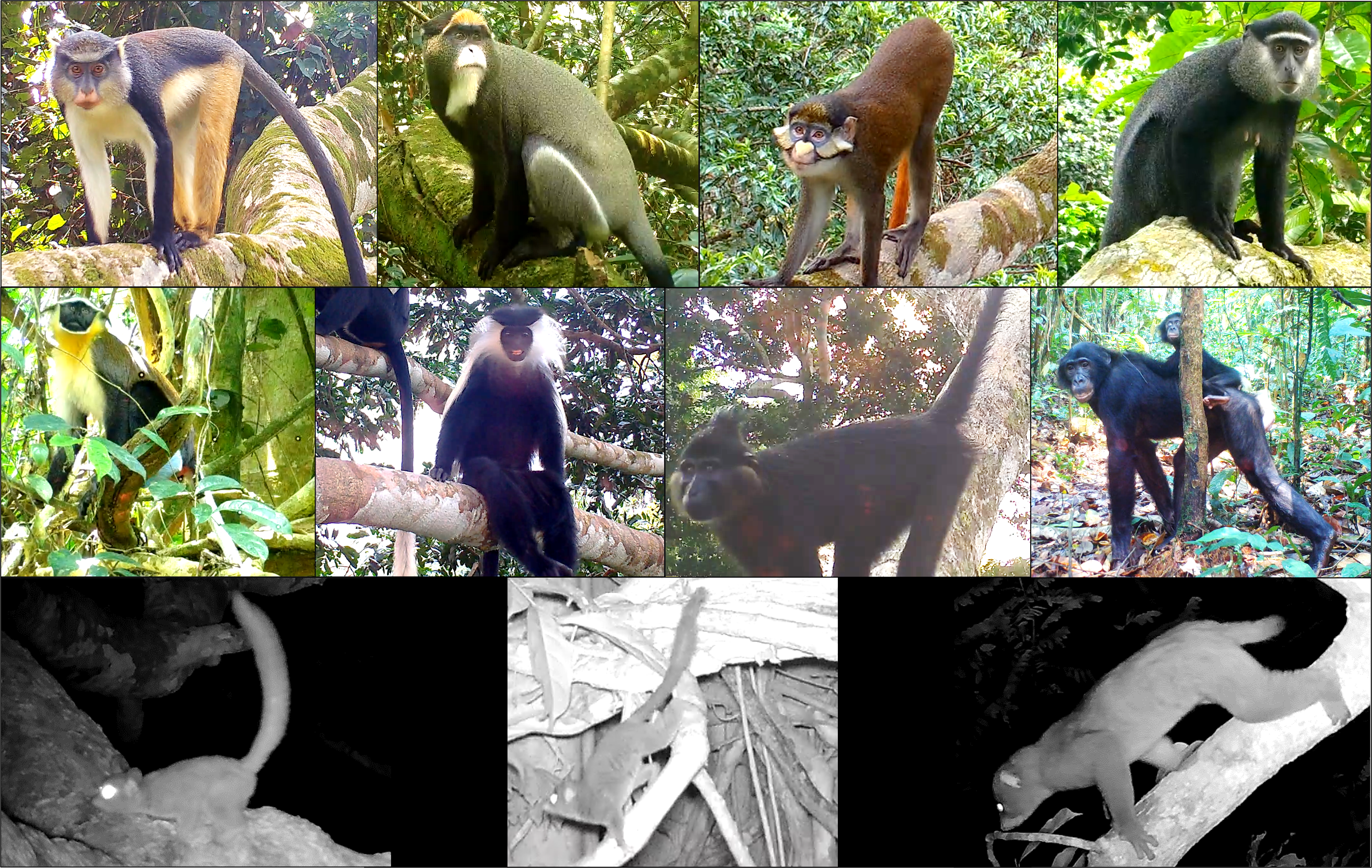

Supplement: gyag047_Supplementary_Data [file gyag047_supplementary_data.zip › Supplementary Data SD4.tif]

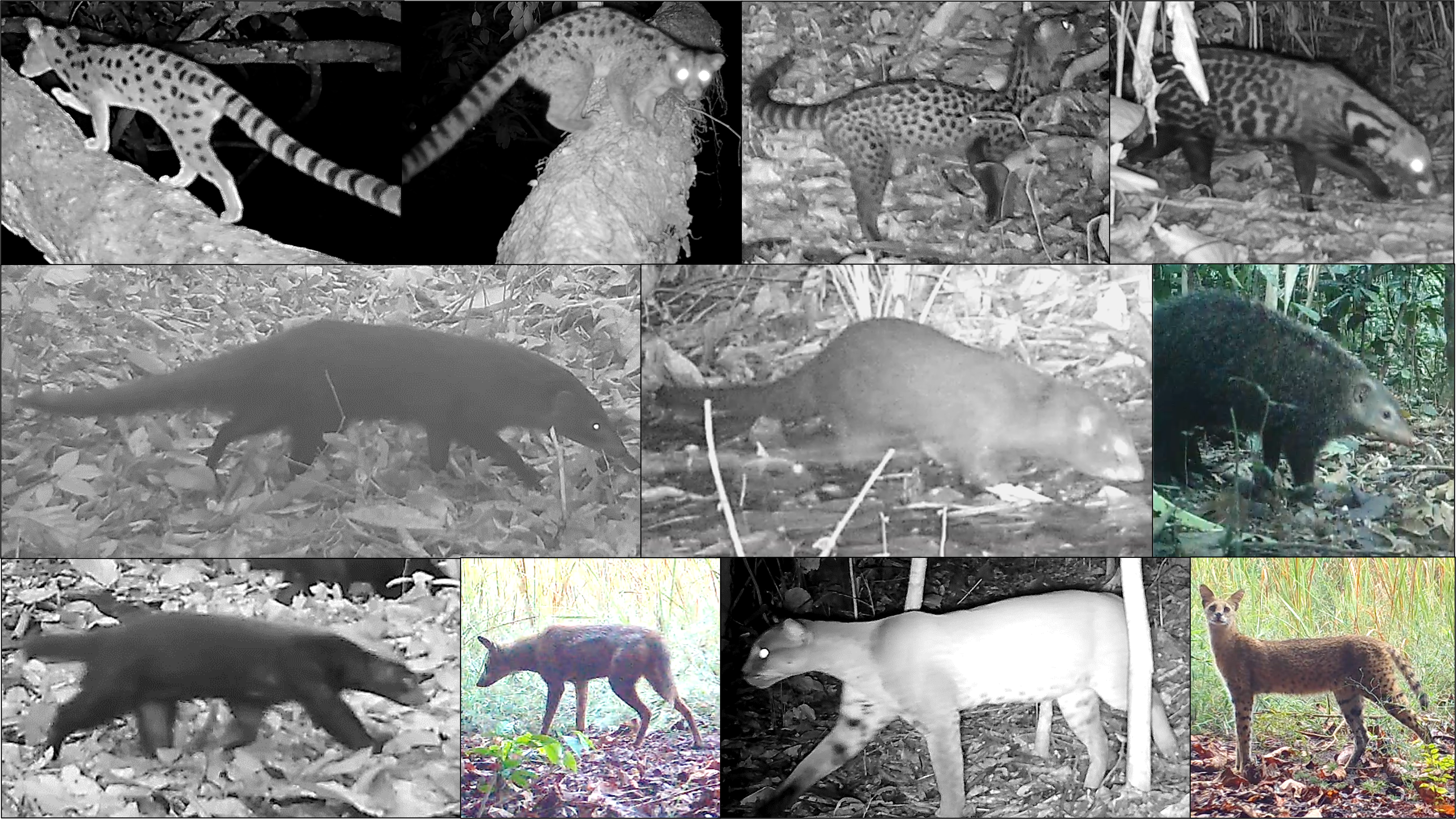

Supplement: gyag047_Supplementary_Data [file gyag047_supplementary_data.zip › Supplementary Data SD5.tif]

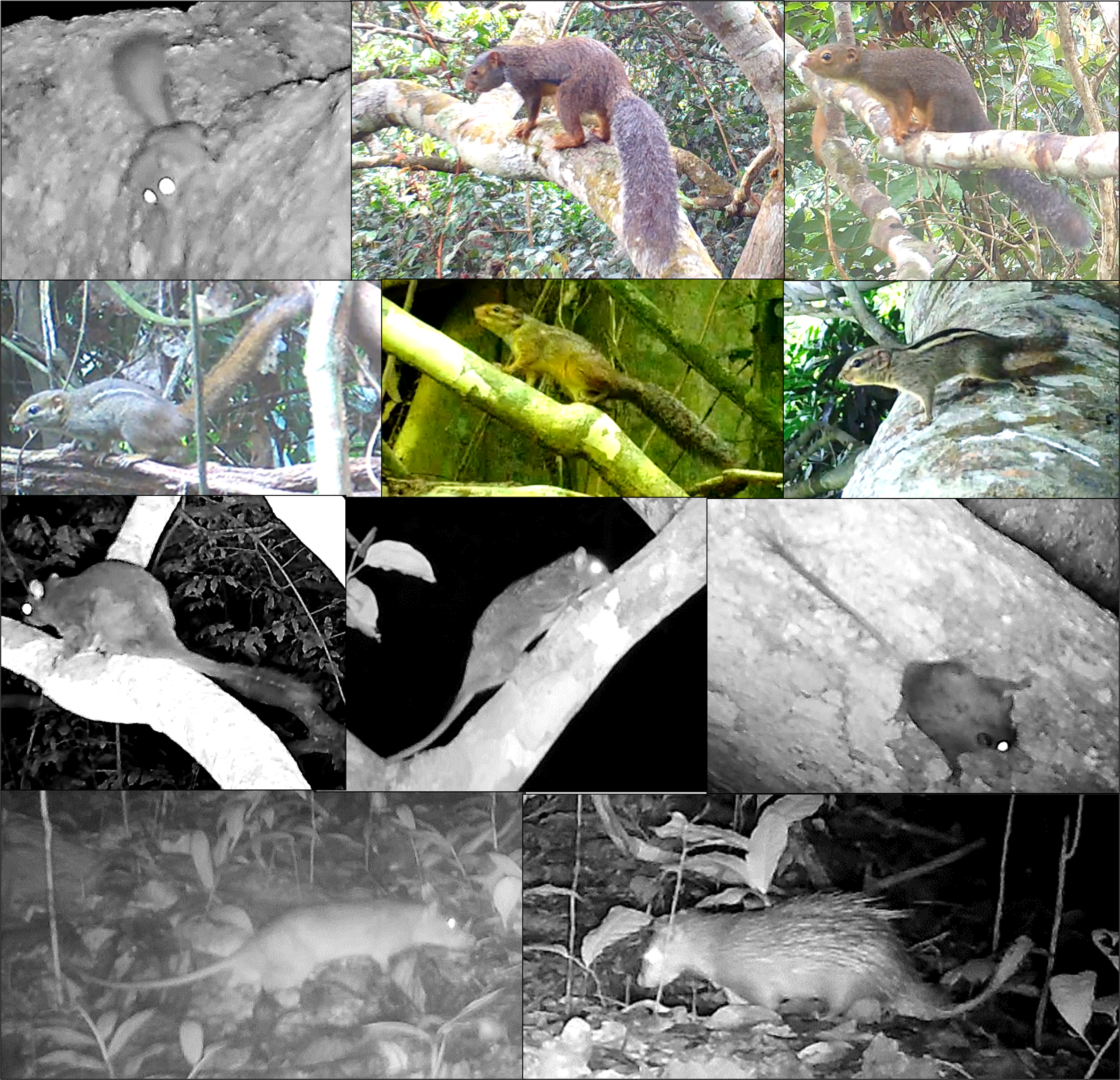

Supplement: gyag047_Supplementary_Data [file gyag047_supplementary_data.zip › Supplementary Data SD6.tif]

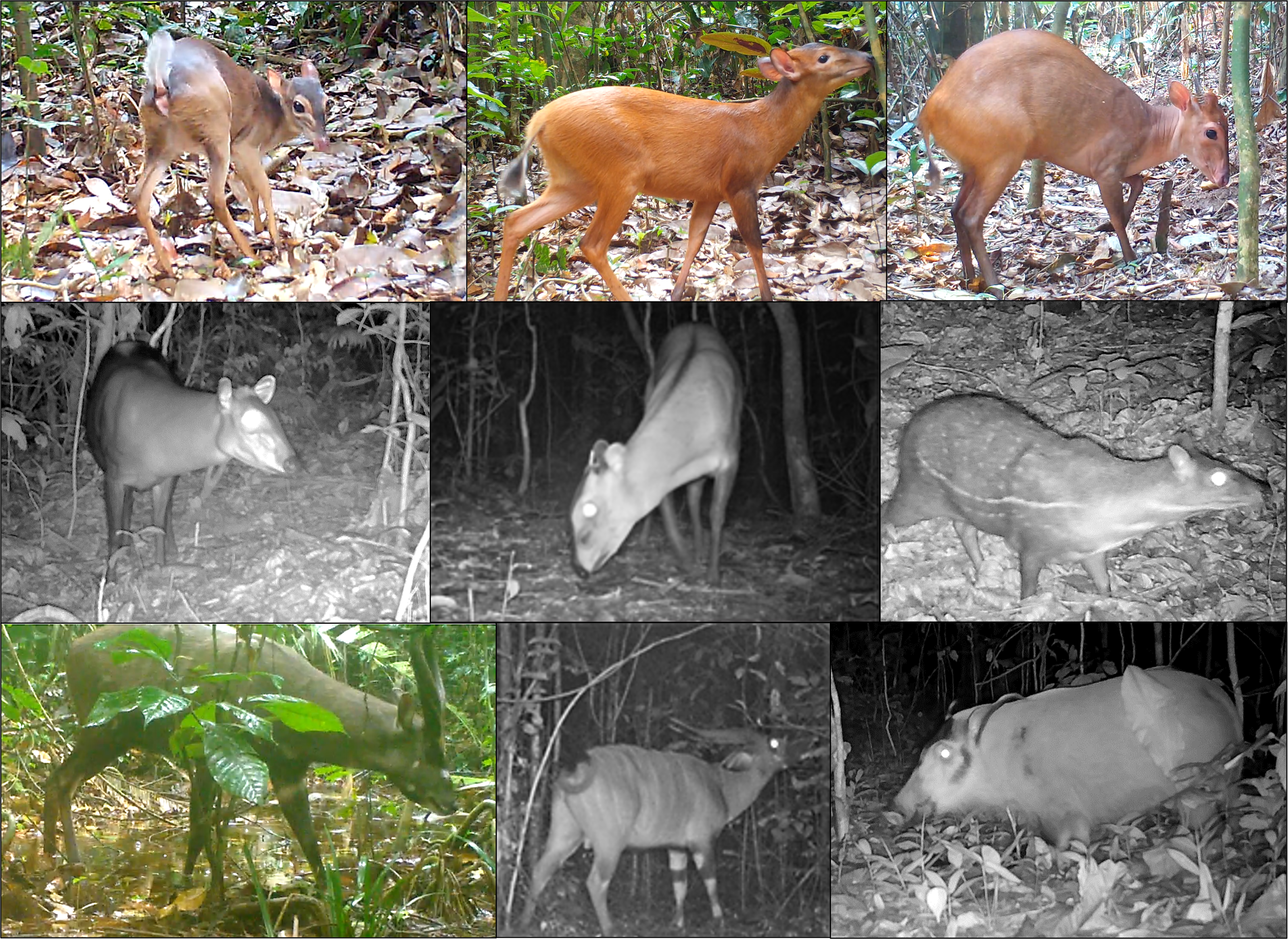

Supplement: gyag047_Supplementary_Data [file gyag047_supplementary_data.zip › Supplementary Data SD7.tif]

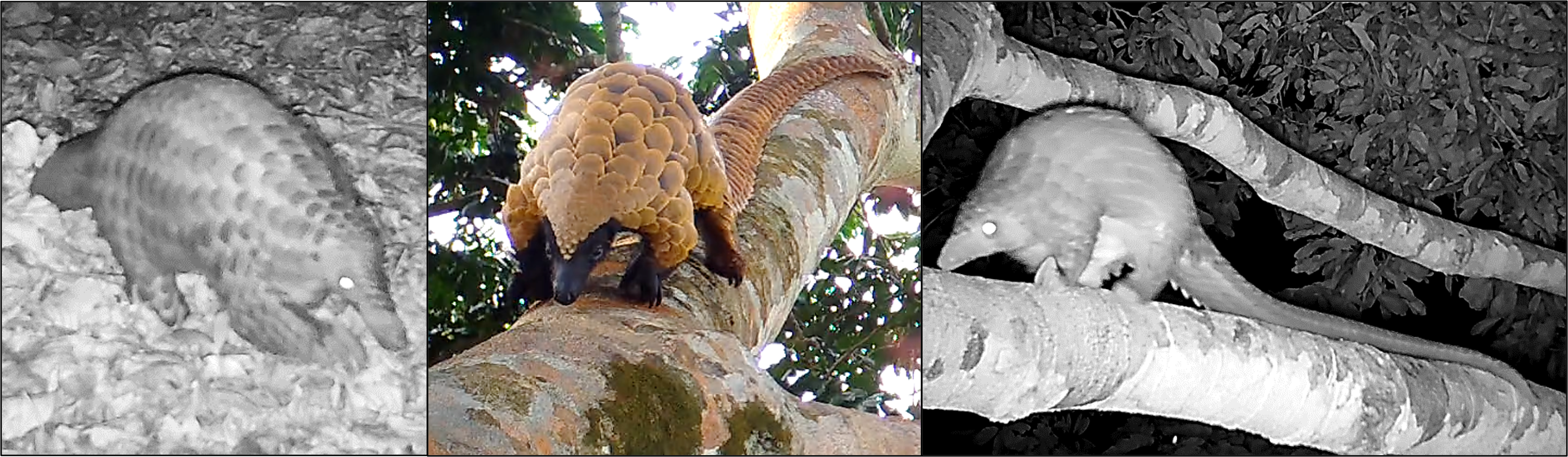

Supplement: gyag047_Supplementary_Data [file gyag047_supplementary_data.zip › Supplementary Data SD8.tif]

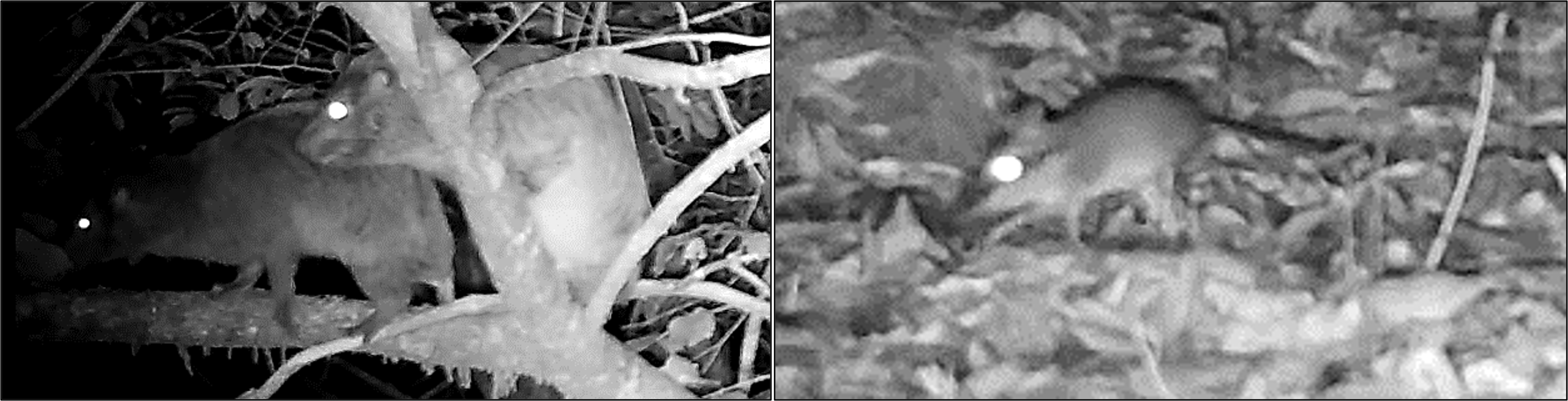

Supplement: gyag047_Supplementary_Data [file gyag047_supplementary_data.zip › Supplementary Data SD9.tif]

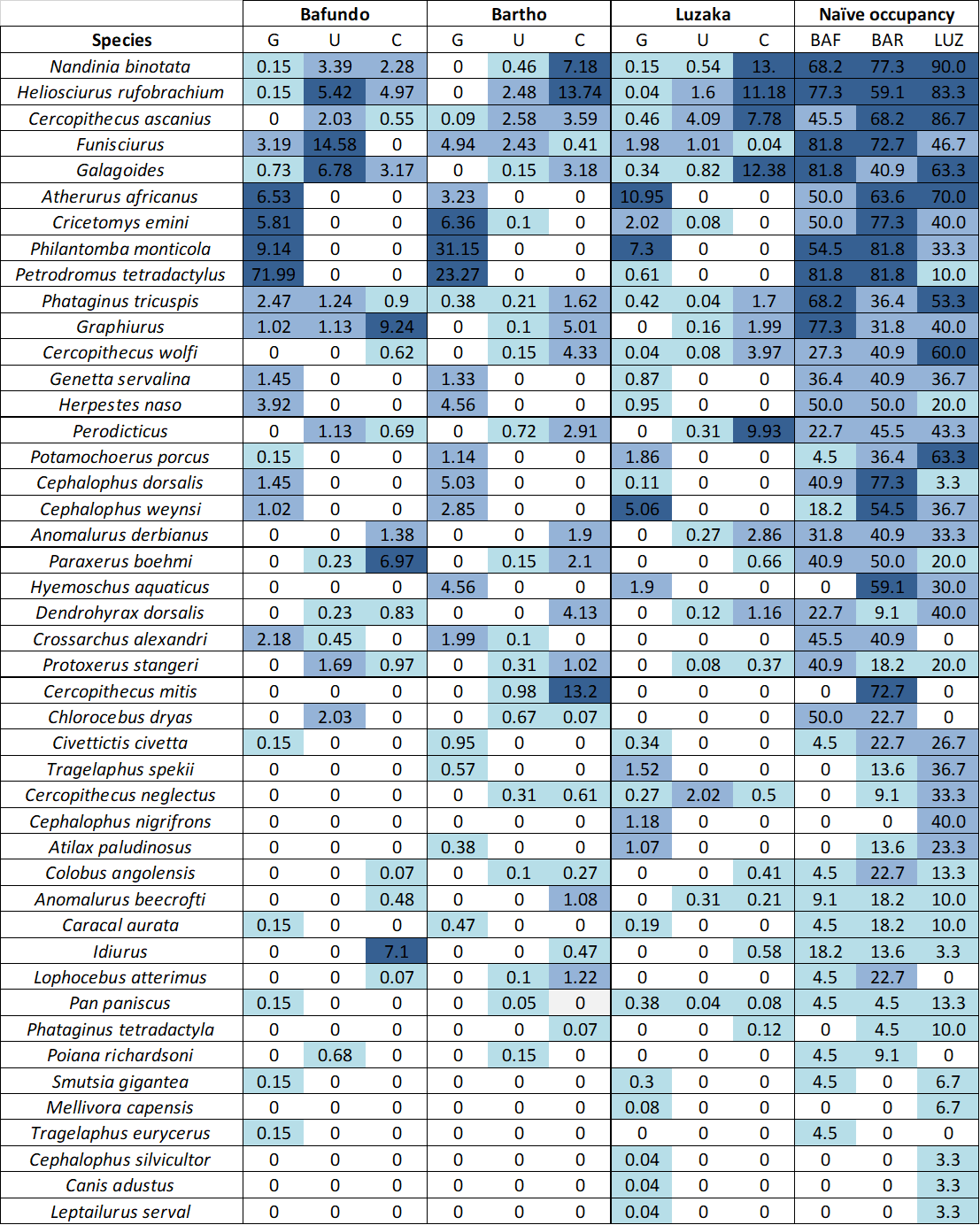

Supplement: gyag047_Supplementary_Data [file gyag047_supplementary_data.zip › Supplementary Data SD10.tif]

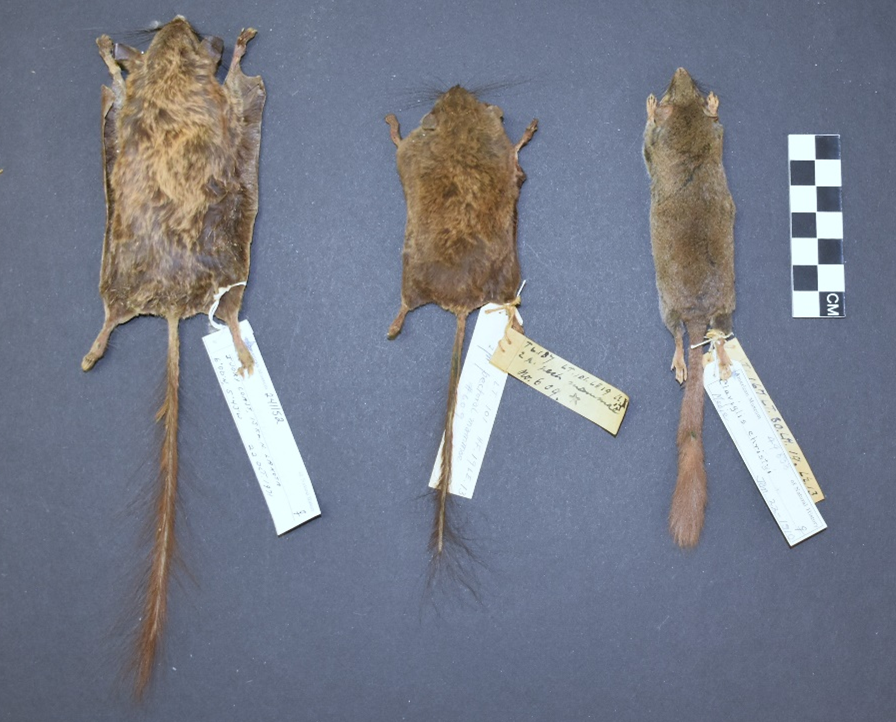

Supplement: gyag047_Supplementary_Data [file gyag047_supplementary_data.zip › Supplementary Data SD11.tif]

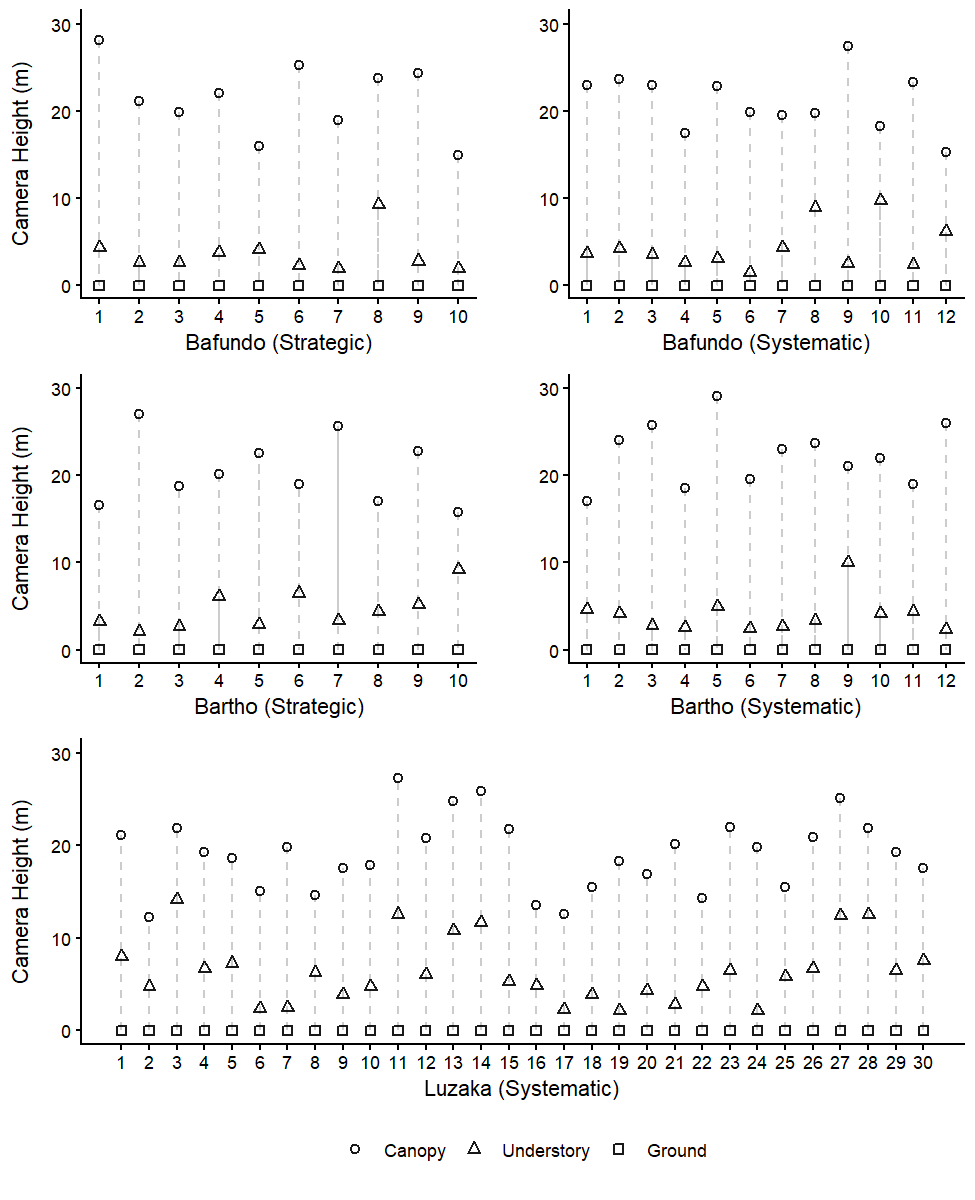

Supplement: gyag047_Supplementary_Data [file gyag047_supplementary_data.zip › Supplementary Data SD1.tiff]
